# Drp1-Dependent Mitochondrial Fission in the Hippocampus Drives Chronic Stress-Induced Depressive-like Behaviors in Mice

**DOI:** 10.3390/ijms27115085

**Published:** 2026-06-04

**Authors:** Qiao Peng, Lijun Ai, Chang Chen, Jiayan Zhang, Qingya Sun, Tiantian Gao, Ming Zhao, Jiarui Zhang

**Affiliations:** 1 School of Integrative Medicine, Nanjing University of Chinese Medicine, Nanjing 210023, China; 2 College of Pharmacy, Guangdong Medical University, Dongguan 523808, China; 3 Jiangsu Collaborative Innovation Center of Chinese Medicinal Resources Industrialization, and National and Local Collaborative Engineering Center of Chinese Medicinal Resources Industrialization and Formulae Innovative Medicine, Nanjing University of Chinese Medicine, Nanjing 220023, Chinamingzhao@njucm.edu.cn (M.Z.); 4 School of Elderly Care Services and Management, Nanjing University of Chinese Medicine, Nanjing 210023, China

**Keywords:** chronic social defeat stress, mitochondrial fission, Drp1, Mdivi-1, depression-like behaviors

## Abstract

The mechanism of action of mice in chronic stress-induced depressive like behavior remains unclear. In this study, we found that chronic social defeat stress (CSDS) upregulates Drp1 expression in mouse hippocampal tissue, leading to excessive mitochondrial fission, which further impairs bioenergetics, induces oxidative stress, disrupts mitochondrial autophagy, and reduces excitatory synaptic transmission. Stereotactic injection of Drp1 inhibitor Mdivi-1 into the hippocampus reversed the aforementioned neuronal defects and alleviated CSDS-induced depressive-like behaviors, including social avoidance, anhedonia, and behavioral despair. Our findings indicate that elevated Drp1 triggers mitochondrial fission, representing a key pathophysiological mechanism underlying stress-induced depression. Therefore, targeting the regulation of mitochondrial dynamics may represent a viable therapeutic strategy.

## 1. Introduction

Currently, Major Depressive Disorder (MDD) is a prevalent and increasingly severe mental illness that imposes a significant social and economic burden worldwide [1]. Despite extensive research, the neurobiological mechanisms underlying MDD remain unclear, and commonly used clinical antidepressants are characterized by a slow onset of action and notable side effects [2]. Chronic stress is considered a major risk factor for depression [3], and animal models such as chronic social defeat stress (CSDS) have been widely used to recapitulate the disease, reflecting key behavioral and neurobiological features.

Mitochondria are important organelles that regulate cellular energy metabolism, calcium homeostasis, and apoptosis signaling. Accumulating evidence suggests that mitochondrial dysfunction is closely associated with the pathophysiology of depression [4]. Postmortem brain studies and preclinical models have consistently reported that alterations in mitochondrial morphology are linked to impaired oxidative phosphorylation in depression [5,6]. Among the various aspects of mitochondrial dynamics, excessive mitochondrial fission has emerged as a key factor in stress-induced neuronal damage [7]. Dynamin-related protein 1 (Drp1) is a key regulator of mitochondrial fission [8]. Upon activation, Drp1 translocates from the cytoplasm to the outer mitochondrial membrane, where it oligomerizes and constricts mitochondria, ultimately leading to mitochondrial fission [9]. Elevated Drp1 expression has been observed in animal models following stress exposure, and both pharmacological and genetic inhibition have been shown to exert neuroprotective effects in various neurological and psychiatric models [10].

The hippocampus, a brain region highly sensitive to chronic stress, plays a critical role in emotion regulation and cognitive function. Studies have shown that patients with depression and stress-exposed animals exhibit structural and functional abnormalities in the hippocampus, including reduced volume and impaired synaptic plasticity [11,12]. At the cellular level, chronic stress also disrupts the mitochondrial dynamics in hippocampal neurons, leading to energy depletion, increased oxidative stress, and synaptic dysfunction [13,14]. However, it remains unclear whether Drp1-mediated mitochondrial fission directly contributes to hippocampal dysfunction in CSDS-induced depression and whether inhibiting this process can restore neuronal homeostasis.

Many recent studies have shown that mitochondrial autophagy, a selective form of autophagy, eliminates damaged mitochondria and is closely linked to mitochondrial quality control and neuronal activity [15]. While impaired autophagy has been associated with the pathogenesis of depression, little is known about the interaction between excessive mitochondrial fission and defective autophagy in the stressed hippocampus [16].

In this study, we used a CSDS mouse model to investigate the role of Drp1-mediated mitochondrial fission in depressive like behavior by examining the morphology and function of hippocampal mitochondria. Additionally, we stereotactically injected the selective Drp1 inhibitor Mdivi-1 into the hippocampus to assess whether inhibiting mitochondrial fission could ameliorate CSDS-induced depressive like behavior, neuronal dysfunction, and impaired mitochondrial autophagy. Our findings indicate that excessive mitochondrial fission leads to hippocampal dysfunction in stress-induced depression and suggest that targeting Drp1-mediated mitochondrial fission may represent a promising therapeutic strategy for alleviating depressive symptoms.

## 2. Results

### 2.1. Exposure to CSDS Induced Depressive-like Behaviors in Mice

**Figure 1:**
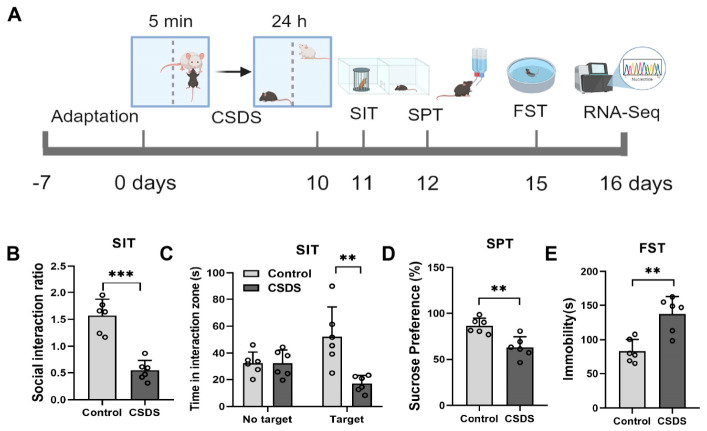
CSDS induces depression-like behaviors in mice. (**A**) Schematic timeline of the chronic social defeat stress (CSDS) paradigm and subsequent behavioral tests, including the social interaction test (SIT), sucrose preference test (SPT), and forced swim test (FST). (**B**) Social interaction ratio in control and CSDS-exposed mice. (**C**) Time spent in the interaction zone during the SIT. (**D**) Sucrose preference measured by the SPT. (**E**) Immobility time recorded during the FST. Data are presented as mean ± SEM (*n* = 6 mice per group). ** *p* < 0.01, *** *p* < 0.001 versus control group (*t*-test or two-way ANOVA).

Following a 10-day CSDS paradigm and subsequent behavioral assessments (Figure 1A), we found that CSDS induced significant social avoidance in mice, as reflected by both the social interaction ratio (Figure 1B) and the time spent in the interaction zone (Figure 1C). Additionally, CSDS-exposed mice exhibited pronounced anhedonia, as evidenced by a significant reduction in sucrose solution intake (Figure 1D), as well as behavioral despair, as indicated by a marked increase in immobility time during the forced swim test (Figure 1E). Taken together, these results demonstrate that CSDS reliably induces depressive-like behaviors in mice.

### 2.2. CSDS Induces Mitochondrial Fission in Hippocampal Neurons of Mice

**Figure 2:**
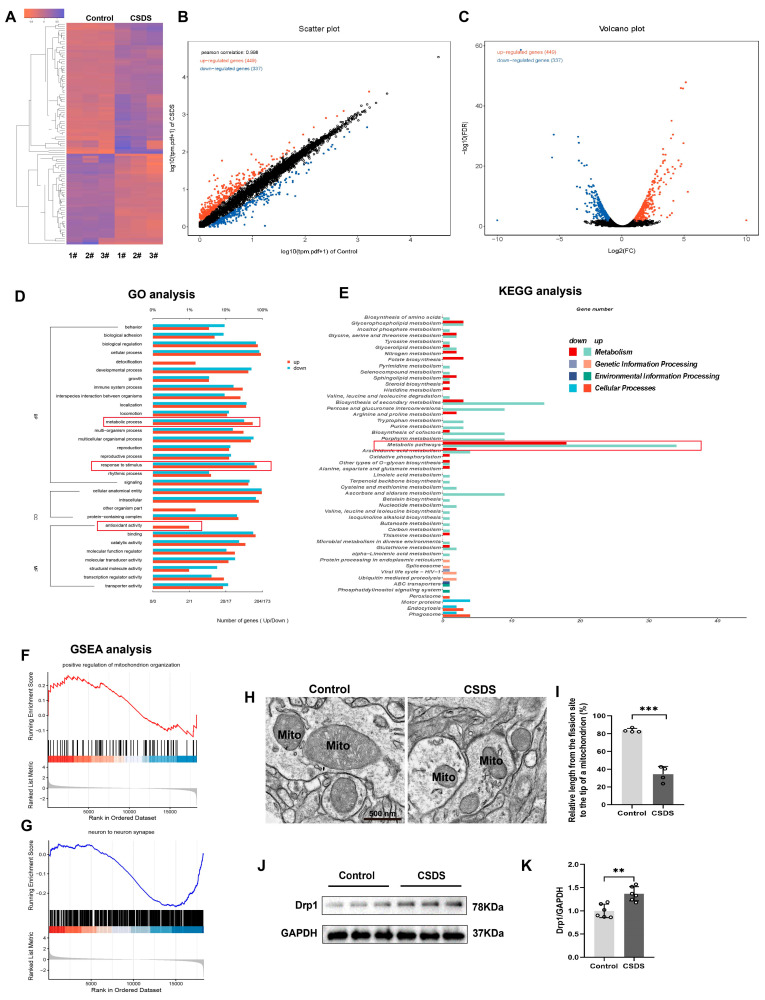
CSDS modeling induced mitochondrial fission in hippocampal neurons of mice. (**A**) The heatmap displays the clustering of gene expression between the control and model groups. (**B**) The volcanic map displays differentially expressed genes (DEGs) in the hippocampus. Red and blue represent upregulated and downregulated genes, respectively. (**C**) Differential genes include upregulation (449) and downregulation. (**D**) Gene Ontology (GO) Enrichment Analysis of DEGs. (**E**) KEGG pathway analysis. (**F**,**G**) Gene Set Enrichment Analysis (GSEA) revealed an increase in mitochondrial activity (**F**) and a decrease in synaptic function (**G**) in mice after CSDS stimulation. (**H**) Representative transmission electron microscopy (TEM) images of mitochondria in hippocampal neurons. Scale bar, 500 nm. (**I**) Calculate the distance variation from the fission site to the mitochondria. (**J**) Representative Western blot of Drp1 protein expression in hippocampal tissue. (**K**) Quantitative analysis and standardized Drp1 levels. The data are mean ± SEM (*n* = 3–6 pre group), ** *p* < 0.01, *** *p* < 0.001 (*t*-test).

To explore the mechanisms underlying depressive-like behaviors induced by CSDS, we performed transcriptomic sequencing of hippocampal tissues. Clustering heatmaps showed clear segregation of gene expression profiles between control and CSDS groups (Figure 2A). The volcano plot showed a linear distribution of differentially expressed genes (DEGs), with 449 upregulated and 337 downregulated (Figure 2B,C). GO analysis further demonstrated that DEGs were enriched in metabolic pathways, response to stress stimuli, and antioxidant activity (Figure 2D). KEGG pathway analysis indicated significant activation of metabolic pathways (Figure 2E). Further GSEA revealed enhanced mitochondrial activity alongside reduced synaptic function in neurons (Figure 2F,G). These findings suggest that both neuronal and mitochondrial integrity may be compromised following CSDS.

Transmission electron microscopy (TEM) was then used to examine mitochondria in hippocampal neurons. CSDS-exposed mice exhibited reduced mitochondrial size (Figure 2H). Quantification of the distance from fission sites to the mitochondrial body confirmed the presence of mitochondrial fission (Figure 2I). Given that DNM1/Drp1 is a key mediator of mitochondrial fission [17], we examined its protein expression in hippocampal tissues. Western blot analysis revealed increased Drp1 protein levels following CSDS (Figure 2J,K), suggesting its involvement in mitochondrial fission. Together, these results indicate that CSDS upregulates Drp1, thereby promoting mitochondrial fission and subsequent mitochondrial dysfunction, which may contribute to stress-induced depression in mice.

### 2.3. Mitochondrial Division Inhibitor Ameliorates CSDS-Induced Depressive-like Behaviors in Mice

Drp1-mediated mitochondrial fission has been reported to play a critical role in stress-induced depression in mice, including the initiation of mitochondrial dysfunction. Mdivi-1, a selective Drp1 inhibitor, has been shown to prevent mitochondrial fission and Bax-mediated mitochondrial outer membrane permeabilization during apoptosis [18]. Previous studies have also demonstrated its antidepressant-like effects, although the underlying mechanisms remain to be fully elucidated [19].

**Figure 3:**
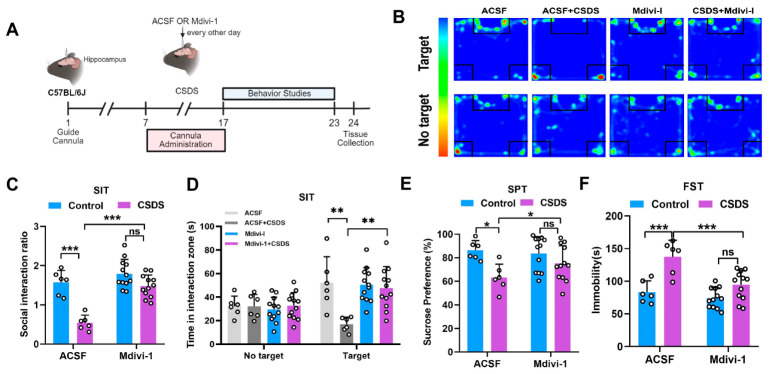
Mdivi-1 ameliorates CSDS-induced depression-like behaviors. (**A**) Schematic diagram of bilateral trocar implantation in the mouse hippocampus and timeline diagram of Mdivi-1 administration and depression like behavior detection. (**B**) Representative track plots of mouse movement during the SIT under four conditions. (**C**) Social interaction ratio in each group. (**D**) Time spent in the interaction zone during the SIT. (**E**) Sucrose preference measured by the SPT. (**F**) Immobility time recorded during the FST. Mdivi-1 (100 μM) or vehicle was infused into the hippocampus every other day. Data are presented as mean ± SEM (*n* = 6 or 12 mice per group), ns, *p* > 0.05, * *p* < 0.05, ** *p* < 0.01, *** *p* < 0.001 (two-way ANOVA).

To investigate the role of Drp1-mediated mitochondrial fission in CSDS-induced behavioral abnormalities, Mdivi-1 (100 μM) or vehicle was bilaterally infused into the hippocampal CA1 region every other day via implanted cannulas (Figure 3A). Behavioral assessments revealed that Mdivi-1 treatment significantly ameliorated CSDS-induced depression-like phenotypes, including social avoidance (Figure 3B), anhedonia, and behavioral despair. Specifically, compared to vehicle-treated CSDS mice, Mdivi-1-treated mice exhibited a higher social interaction ratio and spent more time in the interaction zone during the SIT (Figure 3C,D), showed increased sucrose preference in the SPT (Figure 3E), and displayed reduced immobility time in the FST (Figure 3F). These results indicate that mitochondrial fission contributes to mitochondrial damage and is key to the development of chronic stress-induced depressive-like behaviors in mice.

### 2.4. Mitochondrial Division Inhibitor Ameliorates CSDS-Induced Mitochondrial Dysfunction in the Mouse Hippocampus

**Figure 4:**
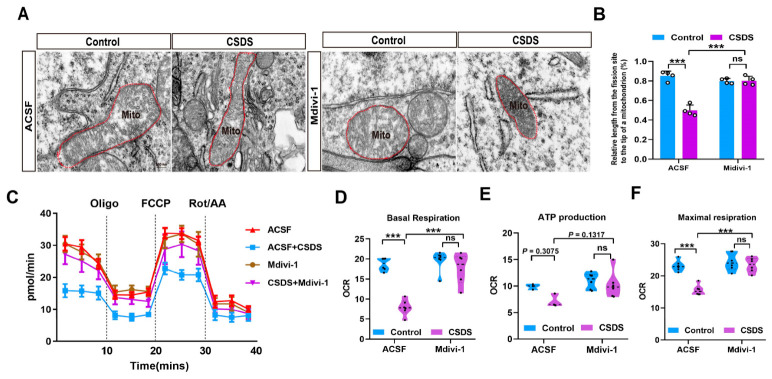
Mdivi-1 rescues CSDS-induced mitochondrial morphological and functional deficits in the hippocampus. (**A**) Representative TEM images of mitochondria in hippocampal neurons from four groups. Scale bar, 200 nm. (**B**) Quantification of the distance from fission sites to the mitochondrial edge. (**C**) Basal respiration measured by Seahorse analysis. (**D**) Maximal respiratory capacity. (**E**) ATP-linked respiration. (**F**) Spare respiratory capacity following FCCP injection. Mdivi-1 (100 μM) was infused into the hippocampus every other day. Data are presented as mean ± SEM (*n* = 4 or 6 mice per group). ns, *p* > 0.05, *** *p* < 0.001 (two-way ANOVA).

TEM was performed to examine mitochondrial morphology in hippocampal neurons. Quantitative analysis of the distance from the fission site to the mitochondrial edge showed that CSDS significantly induced mitochondrial fission. Notably, treatment with the mitochondrial fission inhibitor Mdivi-1 ameliorated CSDS-induced mitochondrial fission (Figure 4A,B).

Furthermore, we used the Seahorse analyzer to evaluate mitochondrial bioenergetic function. Compared with the control mice, CSDS-exposed mice exhibited reduced basal respiration, maximum respiratory capacity, and ATP-related respiration in hippocampal mitochondria (Figure 4C–F), indicating impaired oxidative phosphorylation. Importantly, Mdivi-1 treatment effectively reversed these CSDS-induced mitochondrial functional deficits.

Overall, these results indicate that inhibition of mitochondrial fission enhances mitochondrial respiratory capacity and ameliorates CSDS-induced abnormalities in hippocampal mitochondrial energy metabolism.

### 2.5. Mitochondrial Fission Inhibition Improves CSDS-Induced Hippocampal Neuronal Damage and Oxidative Stress Response in Mice

**Figure 5:**
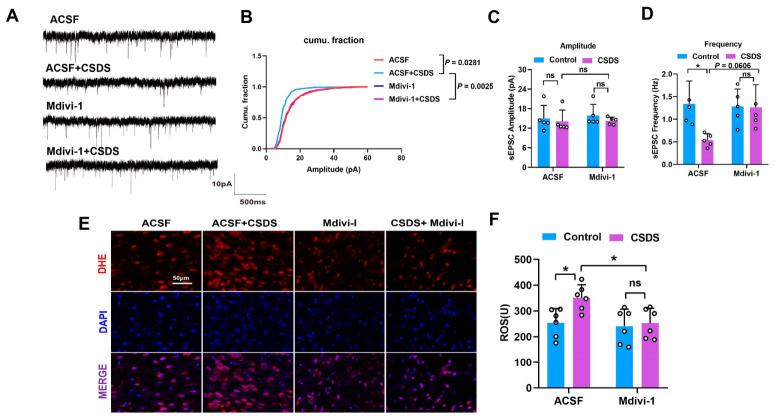
Mdivi-1 alleviates CSDS-induced hippocampal neuronal damage and oxidative stress. (**A**) Representative traces of spontaneous excitatory postsynaptic currents (sEPSCs) recorded from hippocampal neurons in four groups. (**B**) Quantification of sEPSC frequency. (**C**,**D**) Quantification of sEPSC amplitude and frequency. (**E**) Representative dihydroethidium (DHE) staining images of hippocampal sections showing reactive oxygen species (ROS) levels. Scale bar, 50 μm. (**F**) Quantification of DHE fluorescence intensity. Mdivi-1 (100 μM) was infused into the hippocampus every other day. Data are presented as mean ± SEM (*n* = 5 or 6 mice per group). ns, *p* > 0.05, * *p* < 0.05 (two-way ANOVA).

Our transcriptomic data initially showed that CSDS stimulation causes damage to hippocampal neurons in mice. To further evaluate neuronal function, we performed electrophysiological recordings of excitatory synaptic activity in hippocampal neurons. CSDS stimulation significantly reduced the frequency of spontaneous excitatory postsynaptic currents (sEPSCs), while the amplitude remained unchanged. Furthermore, treatment with the mitochondrial fission inhibitor Mdivi-1 rescued the CSDS-induced reduction in sEPSC frequency without affecting the amplitude (Figure 5A–D).

To evaluate the oxidative stress response in hippocampal neurons, dihydroethidium (DHE) staining was used to detect reactive oxygen species (ROS) production. CSDS modeling led to a significant increase in ROS levels in the hippocampus tissue, and Mdivi-1 administration significantly inhibited this effect (Figure 5E,F). Overall, these results indicate that Mdivi-1 treatment ameliorates CSDS-induced hippocampal neuronal damage and oxidative stress response.

### 2.6. Inhibition of Mitochondrial Fission Promotes Mitophagy in Hippocampal Neurons of CSDS Mice

**Figure 6:**
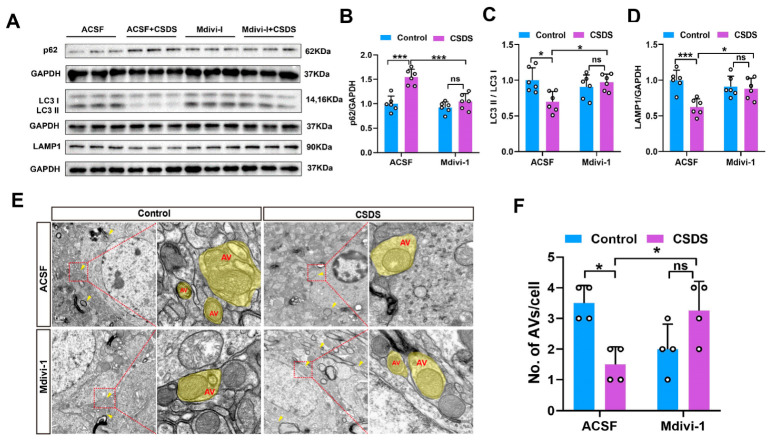
Mdivi-1 restores CSDS-induced impairment of mitophagy in hippocampal neurons. (**A**) Representative Western blots of P62, LC3-I/II, and LAMP1 protein expression in hippocampal tissues from four groups. (**B**) Quantitative analysis of P62 levels normalized to loading control. (**C**) Ratio of LC3-II to LC3-I. (**D**) Quantitative analysis of LAMP1 levels. (**E**) Representative TEM images of autophagosomes in hippocampal neurons. Red letter indicates autophagosomes. Scale bar, 1 μm. (**F**) Quantification of autophagosome numbers per field. Mdivi-1 (100 μM) was infused into the hippocampus every other day. Data are presented as mean ± SEM (*n* = 4 or 6 mice per group). ns, *p* > 0.05, * *p* < 0.05, *** *p* < 0.001 (two-way ANOVA).

Numerous studies have shown that impaired mitophagy is closely related to the pathogenesis of depression [20,21,22]. To evaluate the autophagic status in hippocampal neurons following CSDS, we detected changes in autophagy-related proteins by Western blotting. CSDS modeling was found to increase P62 protein expression, decrease the LC3-II/LC3-I ratio, and reduce LAMP1 protein expression levels, indicating impaired autophagic clearance in neurons. Notably, treatment with the mitochondrial division inhibitor Mdivi-1 reversed these changes (Figure 6A–D), restoring the expression of these autophagy markers to near-control levels.

Consistent with these biochemical findings, TEM revealed a reduction in autophagosome numbers in hippocampal neurons of CSDS-exposed mice, an effect that was significantly ameliorated by Mdivi-1 treatment (Figure 6E,F). Collectively, these results demonstrate that inhibition of mitochondrial fission promotes autophagic responses in mouse hippocampal tissue, thereby alleviating stress-induced depressive-like behaviors.

## 3. Discussion

In this study, we demonstrated that chronic social defeat stress (CSDS) induces depressive-like behaviors in mice, accompanied by excessive mitochondrial fission, elevated Drp1 expression, impaired mitochondrial bioenergetics, and increased oxidative stress. Notably, the selective Drp1 inhibitor Mdivi-1, administered via stereotaxic hippocampal infusion, effectively reversed neuronal functional damage, restored mitochondrial function, promoted mitochondrial autophagy, and alleviated CSDS-induced behavioral deficits. Collectively, our findings suggest that Drp1-mediated mitochondrial fission plays a critical role in stress-induced hippocampal neuronal dysfunction, and suggest that targeting mitochondrial fission may represent a promising therapeutic strategy for the treatment of depression.

Accumulating evidence links mitochondrial dynamics abnormalities to stress-related mental disorders [8,23]. Consistent with previous reports of increased mitochondrial debris in rodent brains following stress exposure [24,25], our TEM analysis revealed that CSDS significantly reduced the mitochondrial size in hippocampal neurons and increased the frequency of fission events. This morphological change was accompanied by an elevation in Drp1 protein levels, the main executor of mitochondrial fission. Although we did not directly assess the post-translational modification of Drp1, which is known to regulate mitochondrial fission [26], the observed increase in total Drp1 expression together with the morphological evidence strongly suggests enhanced fission activity. Mdivi-1, a pharmacological inhibitor of Drp1, alleviated CSDS-induced mitochondrial division and depressive- like behaviors deficits in mice following administration. This finding further supports the view that excessive fission is detrimental to neuronal function under chronic stress conditions.

Recent studies have shown that mitochondrial-derived vesicles (MDVs) are the core of mitochondrial quality control, selectively transporting damaged or specific cargo to lysosomes for degradation [27]. It is worth noting that its biosynthesis is directly regulated by mitochondrial dynamics proteins, which recruit DRP1 to drive the action of MDV [28]. Given that we observed an increase in mitochondrial fission mediated by Drp1 under chronic stress, MDV formation may be simultaneously enhanced. This is particularly important in the central nervous system, where synapses have high energy demands and rely on local mitochondrial function [29]. MDV has been proposed as a carrier for intercellular mitochondrial transfer, with the potential to mechanically transfer functional bioenergy to recipient cells [30]. Therefore, we speculate that stress-induced activation of Drp1 may promote the packaging of oxidative phosphorylation (OXPHOS) components into MDVs, delivering them to synaptic terminals that require energy to maintain local ATP production. Future research needs to explore the biosynthesis of MDVs in chronic stress models and determine whether this pathway represents a protective mechanism against stress-induced synaptic dysfunction.

Normal mitochondrial function is crucial for meeting the high-energy demands of neurons, particularly in brain regions closely associated with stress and emotion regulation, such as the hippocampus [31,32]. Using hippocampal samples for mitochondrial energy metabolism analysis, we found that CSDS inhibited basal respiration, maximal respiration capacity, ATP-linked respiration, and reserve respiration capacity of hippocampal mitochondria. These findings are consistent with clinical reports of reduced mitochondrial oxidative phosphorylation in patients with depression [33,34]. The reversal of these metabolic defects by Mdivi-1 indicates that excessive mitochondrial fission directly impairs bioenergetic function. A potential underlying mechanism is that mitochondrial fragmentation reduces cristae density, which disrupts the assembly of respiratory supercomplexes and ultimately leads to decreased coupling efficiency and ATP synthesis [35,36].

Mitochondria are the main source of reactive oxygen species (ROS) in cells. Using hippocampal tissue slices for DHE staining, we observed a significant increase in ROS production in the hippocampus following CSDS, an effect that was inhibited by Mdivi-1. This finding is consistent with previous studies showing that excessive mitochondrial fission promotes ROS production. Conversely, ROS can activate Drp1, further driving mitochondrial fission, resulting in a deleterious feedforward mechanism [37,38]. Under physiological conditions, moderate ROS production can serve as a signaling molecule; however, sustained oxidative stress can lead to dysregulation of endogenous antioxidant defenses, resulting in lipid peroxidation and DNA damage [39]. By breaking this vicious cycle with Mdivi-1, redox homeostasis can be restored, thereby protecting hippocampal neurons from oxidative damage. Our GO analysis of transcriptome data, which revealed enrichment of terms related to antioxidant activity, provides strong support for the involvement of antioxidant pathways in this process.

Selective removal of damaged mitochondria through mitophagy is a key self-regulation mechanism [40]. Our experimental results showed that CSDS increased P62 expression, reduced the LC3-II/LC3-I ratio and LAMP1 levels, and decreased the number of autophagosomes as observed by TEM, indicating impaired autophagic flux in hippocampal neurons of mice with depressive-like behaviors [41,42]. Concurrently, electrophysiological recordings revealed that CSDS reduced the frequency of sEPSCs without altering their amplitude, suggesting the presence of presynaptic dysfunction [43,44]. Mdivi-1 rescued this change, supporting the notion that mitochondrial fission damages presynaptic function, possibly by impairing ATP supply or calcium buffering [45]. Ultimately, Mdivi-1 enhanced social interaction, increased sucrose preference, and reduced immobility time in depressive-like behaviors, indicating that inhibiting fission alleviates depressive-like behaviors. These effects were achieved through intra-hippocampal delivery, highlighting the important role of hippocampal mitochondrial dynamics in stress-induced abnormalities [46,47].

## 4. Materials and Methods

### 4.1. Animals

6-week-old C57BL/6J mice (male) and 7-month-old CD1 mice (male) were purchased from Cyanen Biosciences (Suzhou, China) [48]. All mice were housed under standardized conditions (12 h light/dark cycle), with room temperature maintained at 22 ± 1 °C and relative humidity ranging from 30% to 80%. Food and water are provided ad libitum. 48 mice were used and randomly assigned to 6 groups: control (*n* = 6), CSDS (*n* = 6), ACSF + Control (*n* = 6), ACSF + CSDS (*n* = 6), Midi-1 + Control (*n* = 12), Midi-1 + CSDS (*n* = 12). All animal procedures were reviewed and approved by the Animal Care Committee of Nanjing University of Chinese Medicine (Approval No. 202409A027) and were conducted in accordance with the guidelines of the National Institutes of Health (NIH), USA.

### 4.2. CSDS Model

The chronic social defeat stress (CSDS) paradigm was performed as previously described [49]. Each male C57BL/6J mouse was placed into a cage containing an aggressive male CD1 mouse for 10 consecutive days. During a daily physical interaction lasting 5 to 10 min, the experimental mice were repeatedly attacked by the CD1 mice and exhibited clear defensive behaviors, such as fleeing or assuming a defensive posture. For the remainder of each 24 h cycle, the two mice were separated by a perforated transparent partition, allowing continuous sensory contact. Subsequent reference to previous literature for mouse behavioral testing [50]. A detailed timeline including the CSDS exposure period (days 1–10), behavioral testing period (days 11–15), and tissue collection (day 16).

### 4.3. Surgical Procedure and Drug Infusion

Prior to hippocampal drug administration, mice were anesthetized by intraperitoneal injection of 1% pentobarbital sodium (50 mg/kg) and then placed in a stereotaxic apparatus (RWD Life Sciences, Shenzhen, China). Bilateral guide cannulas were implanted into the hippocampus (AP −2.1 mm, ML ±2.11 mm, DV −1.65 mm) and secured with dental cement. After surgery, the mice were allowed to recover for seven days. Subsequently, each mouse received microinjection of Mdivi-1 (mitochondrial fission inhibitor, MCE, South Brunswick, NJ, USA, Catalog number HY-15886, 100 μM) into the hippocampus, with artificial cerebrospinal fluid (ACSF) as a vehicle control. All infusions were performed using a microinfusion pump (Harvard Instruments, Shanghai, China) at a rate of 0.1 μL/min.

### 4.4. Social Interaction Test

Before each test and between trials, the apparatus was cleaned with 75% ethanol. The test was conducted in a 44 cm × 44 cm open field containing a 10 × 6 cm wire mesh cage. The entire session lasted 5 min, consisting of a 2.5 min habituation phase (without a CD1 mouse) followed by a 2.5 min testing phase (with a CD1 mouse). An interaction zone of 14 cm × 26 cm was defined around the wire mesh cage. The social interaction (SI) ratio was calculated as follows: (time spent in the interaction zone during the testing phase)/(time spent in the interaction zone during the habituation phase) × 100%.

### 4.5. Sucrose Preference Test

The test consisted of three phases. The mouse adaptation period is 24 h, using two bottles containing 2% sucrose or water; the second phase was a 24 h period of food and water deprivation; the third phase was the testing time, which lasted 8 h, and the water and sucrose bottles were switched at the fourth hour. The percentage of sucrose preference using the formula: (sucrose intake/total intake) × 100%.

### 4.6. Forced Swimming Test

A transparent acrylic (Plexiglas) cylinder (approximately 30 cm in height and 15 cm in diameter) was filled with warm water (25 ± 1 °C) to a depth of approximately 15 cm. Behavioral despair was evaluated using the forced swimming test. Mice were tested individually for 5 min. The immobility time of each mouse was recorded, with immobility defined as floating without movement except for the minimal movements necessary to maintain buoyancy. Immobility time was automatically measured by the ANY-maze video-tracking system (Stoelting Co., Wood Dale, IL, USA).

### 4.7. Mitochondrial Respiration Assay

Mitochondria were isolated from mouse hippocampal tissue using a reagent kit (Beyotime, Shanghai, China). The purity and functionality of the isolated mitochondria using this kit have been confirmed [51]. Oxygen consumption was then measured using a Seahorse XFp analyzer (Agilent, Santa Clara, CA, USA). Mitochondrial respiration- related parameters were evaluated by adding 10 mM succinate and 2 mM fisetin to the isolated mitochondria in a well plate, followed by sequential injection of 4 mM ADP, 3 mM oligomycin, 4 mM FCCP, and 4 mM antimycin A.

### 4.8. RNA Sequencing

#### 4.8.1. RNA Extraction and Purification

Hippocampal tissue was collected into a 1.5 mL EP tube, and TRIzol (1.5 mL) was added. The sample was mechanically homogenized by grinding for 30 s, followed by incubation for 5 min. The lysate was cleared by centrifugation (12,000× *g*, 5 min, 4 °C) and then mixed with chloroform/isoamyl alcohol (24:1 ratio). After further centrifugation at 12,000× *g* for 8 min at 4 °C, the aqueous phase was collected. RNA was precipitated with 0.67 volumes of isopropanol at −20 °C for ≥2 h, followed by another centrifugation. The RNA pellet was washed with 75% ethanol, dried, and dissolved in DEPC-treated water.

#### 4.8.2. Library Preparation and Sequencing

Total RNA quality was assessed prior to mRNA capture using oligo(dT) beads. The captured mRNA was fragmented and reverse-transcribed into double-stranded cDNA. After end repair and A-tailing, adapters were ligated, followed by PCR amplification to construct the libraries. Qualified libraries were circularized, and residual linear DNA was removed. The circular DNA was then amplified by rolling circle amplification to form DNA nanoballs (DNBs), which were loaded onto a flow cell and sequenced using combinatorial Probe-Anchor Synthesis (cPAS).

### 4.9. Transmission Electron Microscopy

Mice were anesthetized by intraperitoneal injection of pentobarbital sodium (50 mg/kg) and then sequentially perfused via the heart with physiological saline (20 mL, 37 °C), fixative (20 mL, 0.25% glutaraldehyde in 0.1 M phosphate buffer, 37 °C), and 0.25% glutaraldehyde (100 mL, 4 °C). The brains were dissected and placed in fixative overnight at 4 °C. Hippocampal slices of 100 μm thickness were then prepared using a Leica VT1000S vibratome (Leica Biosystems, Nussloch, Germany), fixed with 1% osmium tetroxide (OsO_4_) for 30 min at room temperature, dehydrated in graded ethanol, and finally embedded in resin. Ultrathin sections of 50 nm thickness were cut using a microtome, picked up onto a nickel grid, and observed under an electron microscope (Zeiss EM900, Oberkochen, Germany).

### 4.10. Western Blot Analysis

Mouse hippocampal tissue was collected and homogenized in RIPA buffer containing 1% PMSF and protease inhibitor (Roche, Basel, Switzerland, 11873580001) to extract protein. The protein concentration was determined using a BCA assay (Thermo Fisher Scientific, Waltham, MA, USA, 23225). 20 µg of total protein were separated by SDS-PAGE and transferred onto a PVDF membrane. The membranes were blocked with 5% skim milk, incubated overnight with primary antibody (Drp1, Proteintech, Rosemont, IL, USA, 12957-1-AP, 1:1000; P62, 31403-1-AP, 31403-1-AP, 1:3000; LC3-I/-II, Cell Signaling Technology, Danvers, MA, USA, #12741, 1:1000; LAMP1, Proteintech, 32435-1-AP, 1:1000 and GAPDH, Proteintech, 60004-1-Ig, 1:5000) at 4 °C, and then incubated with HRP-conjugated secondary antibody (Cell Signaling Technology, 7074S, 7076S, 1:10,000) 1 h at room temperature. Signals were detected via chemiluminescence and captured using the Tanon 4600 system (Tanon Science & Technology Co., Ltd., Shanghai, China). All protein band intensities were normalized to GAPDH as a loading control.

### 4.11. Electrophysiology

According to a previous report [19], following isoflurane anesthesia, mice were perfused with 30 mL of oxygenated ice-cold dissection buffer (in mM: 110 choline chloride, 25 NaHCO_3_, 2.5 KCl, 1.25 NaH_2_PO_4_, 0.5 CaCl_2_, 7 MgSO_4_, 25 D-glucose, 11.6 sodium ascorbate, 3.1 sodium pyruvate). Hippocampal slices were collected in artificial cerebrospinal fluid (ACSF, in mM: 126 NaCl, 2.5 KCl, 26 NaHCO_3_, 2 CaCl_2_, 2 MgCl_2_, 1.25 NaH_2_PO_4_, 10 D-glucose) at 35 °C for 30 min and then maintained at room temperature. For sEPSC recording, slices were transferred to a perfusion chamber on an Olympus microscope. Patch electrodes were filled with an internal solution containing potassium gluconate, HEPES, Mg^2−^ ATP, and Na_3_-GTP (pH 7.2, 295–300 mOsm).

### 4.12. DHE Staining

After deep anesthesia, the mouse brains were dissected and frozen. Sections of 12 μm thickness were then prepared and mounted onto glass slides. The sections were incubated with dihydroethidium (DHE, 2 μmol/L in PBS). Fluorescence images were captured under a microscope (Olympus, Hachioji-shi, Japan) using a 20× objective lens, with at least six images collected per slide. The average fluorescence intensity, reflecting superoxide levels, was quantified using ImageJ (version 1.54c, National Institutes of Health, USA).

### 4.13. Statistical Analysis

RNA-seq reads were trimmed with Trimmomatic (v0.39) and analyzed with DESeq2 (v1.34.0). Differentially expressed genes were defined by FDR < 0.05 and |log2(fold change)| ≥ 1. Functional enrichment analysis was performed using cluster Profiler (v4.2.2). GraphPad Prism 8.0 (GraphPad Software, San Diego, CA) was used for statistical analysis. Before conducting parametric tests, normality was assessed using the Shapiro–Wilk test, and homogeneity of variance was assessed using Levene’s test. When data met both assumptions (*p* > 0.05), a two-tailed Student’s *t*-test (for two groups) or a one-way/two-way ANOVA (for multiple groups) was performed. Statistical significance was set at *p* < 0.05. All data are presented as mean ± standard error of the mean (SEM).

## 5. Conclusions

In summary, our study suggests that CSDS upregulates Drp1 expression and promotes excessive mitochondrial fission in hippocampal neurons, leading to bioenergetics impairment, oxidative stress, mitophagy defects, and presynaptic dysfunction. Pharmacological inhibition of Drp1 with its effective inhibitor Mdivi-1 reverses these abnormalities and ultimately ameliorates depressive-like behaviors in mice. These findings confirm that Drp1-mediated mitochondrial fission is a key pathophysiological mechanism underlying stress-induced depressive-like behaviors in mice and suggest that regulating mitochondrial dynamics and quality control may provide new avenues for therapeutic interventions.

## Data Availability

The data presented in this study are available on request from the corresponding author due to the large volume of raw data.
